# Dietetical and Medical Hydrology

**Published:** 1850-07

**Authors:** 


*Dietetical and Medical Hydrology. A Treatise on Baths; including cold, sea, warm, hot, vapor, gas, and mud Baths; also, on the watery regimen, Hydropathy, and Pulmonary Inhalation; with a description of Bathing in ancient and modern times.* By John Bell, M. D., etc., etc., etc. Philadelphia: Barrington & Haswell, 1850. 12 mo., pp. 658.

This volume may be appropriately styled a cyclopædia of therapeutical and hygienic Hydrology. The learned author treats of water under all the various aspects in which it becomes a prophylactic and remedial agent, and has collected what relates to the subject in medical literature from Hippocrates to the present day. The work appears opportunely, now that the employment of water in medical practice is receiving more consideration than it has done for some years past. We do not doubt that, as an important element in the prevention and cure of disease, it has fallen into undeserved neglect. That it has been pressed into the service of quackery, under the denomination of *Hydropathy*, tends to create a prejudice in the minds of many. But it is a remedy legitimately belonging to medicine, and to appreciate its importance, and become acquainted with the various modes and conditions in which it is applicable, the medical inquirer is under no necessity to go without the pale of the literature of the Profession. Dr. Bell's treatise affords ample evidence of the correctness of this remark.

Although we may not concur fully with the learned author in all his views, the work is, we think, calculated to render valuable service, by directing the attention of the Profession to the subject, and thereby aiding to protect from the extravagances of a popular delusion, an agent sufficiently potent for much evil in the hands of the ignorant, and one which, directed with discrimination and judgment, may be made more available for useful ends than it now is in medical practice. The work is for sale at the bookstore of G. Derby & Co.

